# MiR-302a sensitizes leukemia cells to etoposide by targeting Rad52

**DOI:** 10.18632/oncotarget.17878

**Published:** 2017-05-16

**Authors:** Xiaoning Liu, Chun Heng, Yuanyuan Li, Liang Yu

**Affiliations:** ^1^ Central Laboratory, Huai′an First People’s Hospital, Nanjing Medical University, Huai’an, Jiangsu Province, 223300, China; ^2^ Hematology Department, Huai’an First People’s Hospital, Nanjing Medical University, Huai’an, Jiangsu Province, 223300, China

**Keywords:** miR-302a, acute myeloid leukemia (AML), VP-16 resistance, Rad52

## Abstract

miR-302a have been reported to participate in various physiological and pathological processes, however, a role for miR-302a in etoposide (VP-16) resistance of acute myeloid leukemia (AML) has not been reported. In this study, the aberrant expression of miR-302a was analyzed in patients with AML and in the AML HL-60 and U937 cell lines. Overexpression of miR-302a, by targeting the 3′UTR of Rad52, enhanced VP-16 sensitivity in the HL-60 and U937 cell. Accordingly, knockdown of Rad52 sensitized the HL-60 and U937 cells to VP-16-induced apoptosis and proliferation suppression. In addition, miR-302a enhanced the tumor-suppressive effect of VP-16 in a xenograft model of human HL-60 and U937 cell lines. Moreover, miR-302a repressed the AKT/Gsk3β/β-catenin pathway after Rad52 inhibition. Reintroduction of Rad52 reversed miR-302a-induced signaling suppression. The results of the present study demonstrated that miR-302a may be a target for the treatment of AML and a potential indicator of the therapeutic sensitivity of AML to VP-16.

## INTRODUCTION

Acute myeloid leukemia (AML) is the most prevalent adult leukemia in China. AML, considered as incurable, but now cured in approximately 35%–40% of patients [1]. For those >60 years old, the prognosis remains grim [2]. Chemotherapy is still considered one of the main therapies in AML treatment. Etoposide (VP-16) is an agent that targets DNA topoisomerase II and causes DNA single- and double-strand breaks [3, 4]. It has been widely used for the treatment of many types of cancers including AML [5, 6, 7]. Unfortunately, most patients who respond to primary chemotherapy later experience relapse. Therefore, identification of novel diagnostic markers and therapeutic targets is crucial.

MicroRNAs (miRNAs) are a class of non-coding RNAs composed of approximately 22 nucleotides which function primarily to regulate protein expression at post-transcriptional level by specifically binding to the 3’-untranslated region (3’-UTR) of mRNAs. A large number of studies have shown that miRNAs are involved in hematopoiesis and leukemogenesis and linked to the development of resistance against chemotherapy in many cancers [8, 9]. It has been reported that miR-302a involves in maintaining stemness of hESCs [10, 11, 12], suppressing tumor cell proliferation and inducing cancer cell apoptosis by directly targets Rad52 and AKT1 [13]. However, there is still little known about the potential role of miR-302a in VP16-based AML chemotherapy and further research is needed.

RAD52 was first identified in yeast and playing an important role in repair of DNA double-strand breaks at active transcription sites during the S phase of the cell cycle [14, 15]. The frequent microRNA-induced increase or deficiency of RAD52-mediated DNA repair due to microRNA binding alterations likely contributes to either the prevention or progression of breast, brain, or liver cancers [16].

In this study, the potential alterations in the expression pattern of miR-302a were evaluated in AML patient and in the AML cell lines. We found that overexpression of miR-320a led to elevated VP-16 sensitivity. We also identified the potential value of miR-302a in AML cell lines. The mechanism is direct regulation of RAD52 by miR-302a, leading to regulation of the intrinsic AKT/Gsk3β/β-catenin pathway in AML. miR-302a may serve as a therapeutic target or diagnostic/prognostic marker for leukemia therapy.

## RESULTS

### Reduced expression of miR-302a in AML patients and cell lines

**Figure 1:**
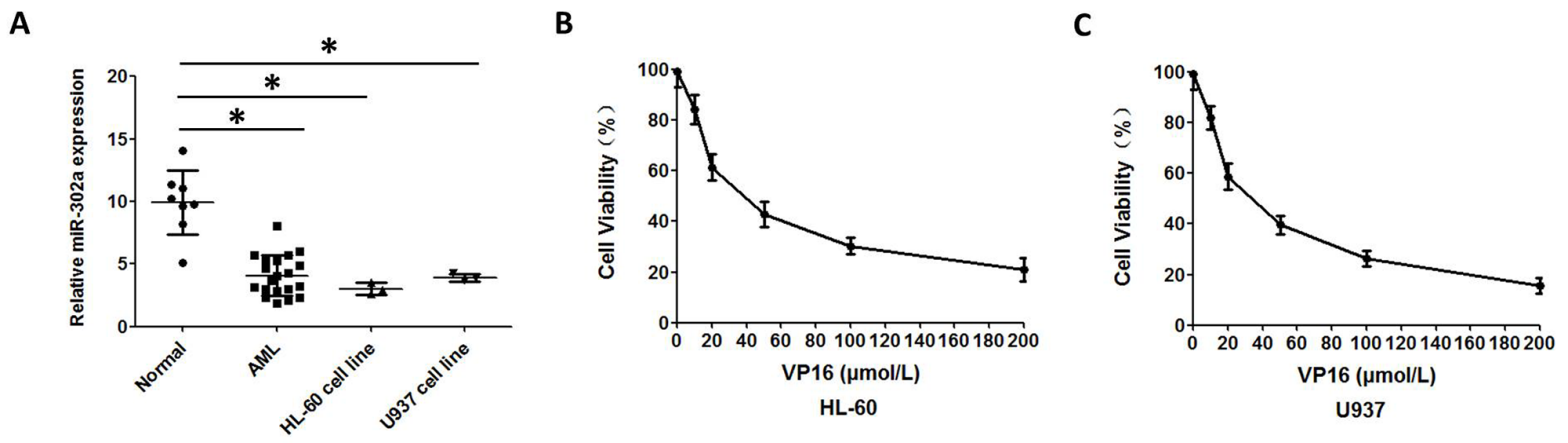
Reduced expression of miR 302a in AML patients and AML cell lines **(A)** qRT-PCR analysis of miR-302a expression in MNCs from healthy people, AML patients and AML cell lines. **(B)** the proliferation of HL-60 treated with different concentration of VP16 measured by CCK8. **(C)** the proliferation of U937 treated with different concentration of VP16 measured by CCK8. Each result represents the mean of three independent experiments.* P<0.05, ** P<0.01.

In order to investigate potential alterations in the expression pattern of miR-302a in AML, the expression levels of miR-302a were measured by qRT-PCR in 20 patients with AML and in the AML HL-60 and U937 cell lines, then compared with the expression levels of miR-302a in 8 healthy volunteers. Interestingly, miR-302a was significantly down-regulated in leukemic cells in both primary AML samples and cell lines, as compared with that of MNCs (Mononuclear cells) isolated from the peripheral blood of healthy volunteers (Figure 1A). Next, we assessed the functional role of VP-16 in proliferation of the HL-60 and U937 cell lines. The growth inhibition curve was shown in Figure 1B and Figure 1C.

### Overexpression of miR-302a enhances the sensitivity to VP-16 in AML cell lines

**Figure 2:**
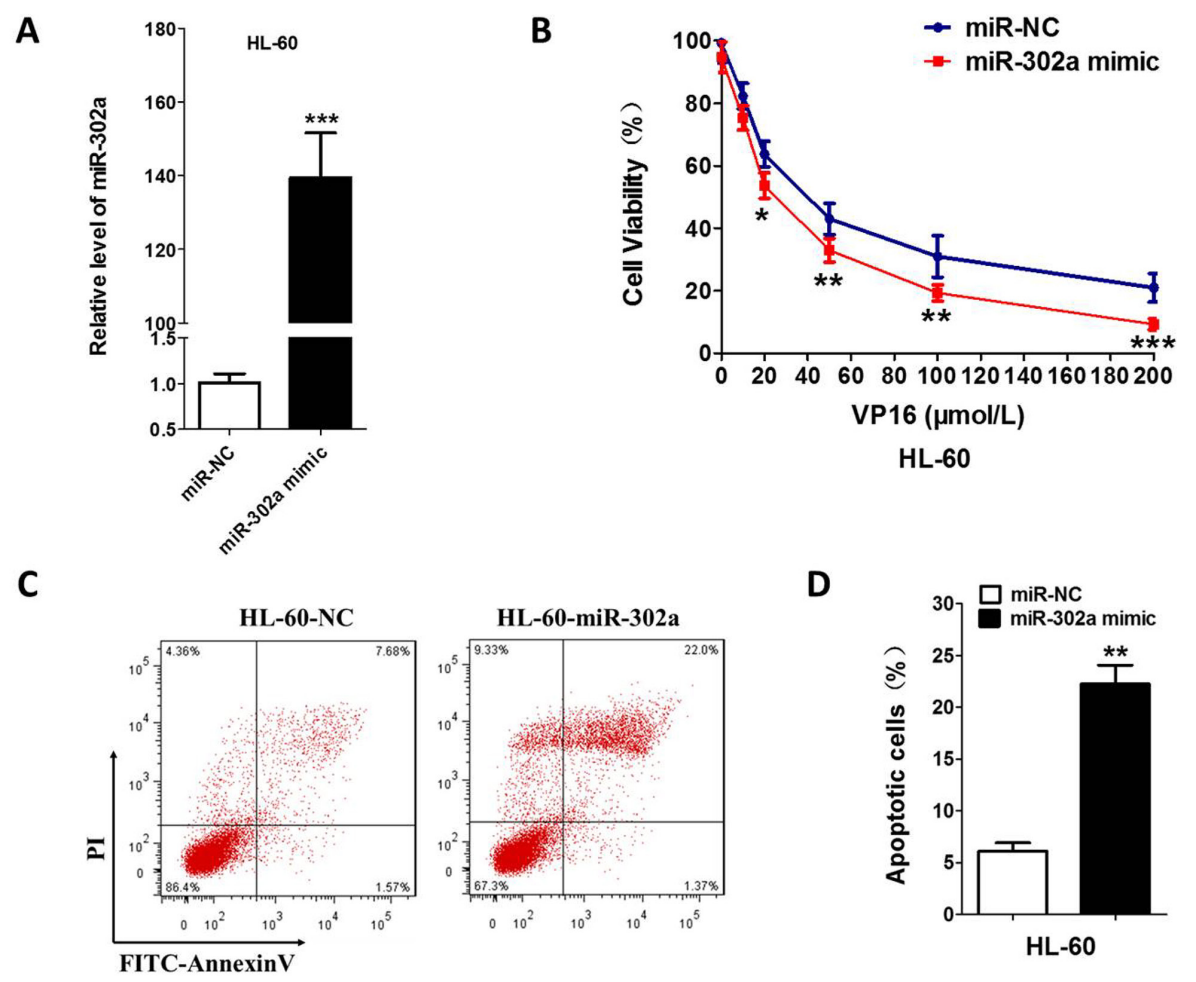
Overexpression of miR-302a enhances the sensitivity to VP-16 in HL-60 Cell **(A)** HL-60 was transfected the miR-302a or negative control by Lipo2000. the expression level of miR-302a was measured by qRT-PCR. **(B)** compare to negative control, overexpression of miR-302a enhance the sensitivity to VP16 measured by MTT in HL-60. **(C,D)** cells were stained with PI and FITC-Annexin V, the percentage of apoptosis cell measured by flow cytometry. Each result represents the mean of three independent experiments.* P<0.05, ** P<0.01. *** P<0.001.

In order to investigate the VP-16 sensitivity of the AML cell line influenced by miR-302a, we transiently transfected the HL-60 and U937 cells with miR-302a mimic or scrambled control. Increased expression of miR-302a upon transfection were determined by qRT-PCR (Figure 2A and Supplementary Figure 1). After 48 h of transfection, MTT assays showed that overexpression of miR-302a led to enhanced inhibition of proliferation in comparison with the control (Figure 2B and Supplementary Figure 1). Compared with the control cells, the apoptosis induced by VP-16 was increased by ∼15% in the HL-60-miR-302a cells (Figure 2C, 2D and Supplementary Figure 1), which indicated that miR-302a promotes the apoptosis of HL-60 and U937 cells.

### Rad52 is a direct target of miR-302a

**Figure 3:**
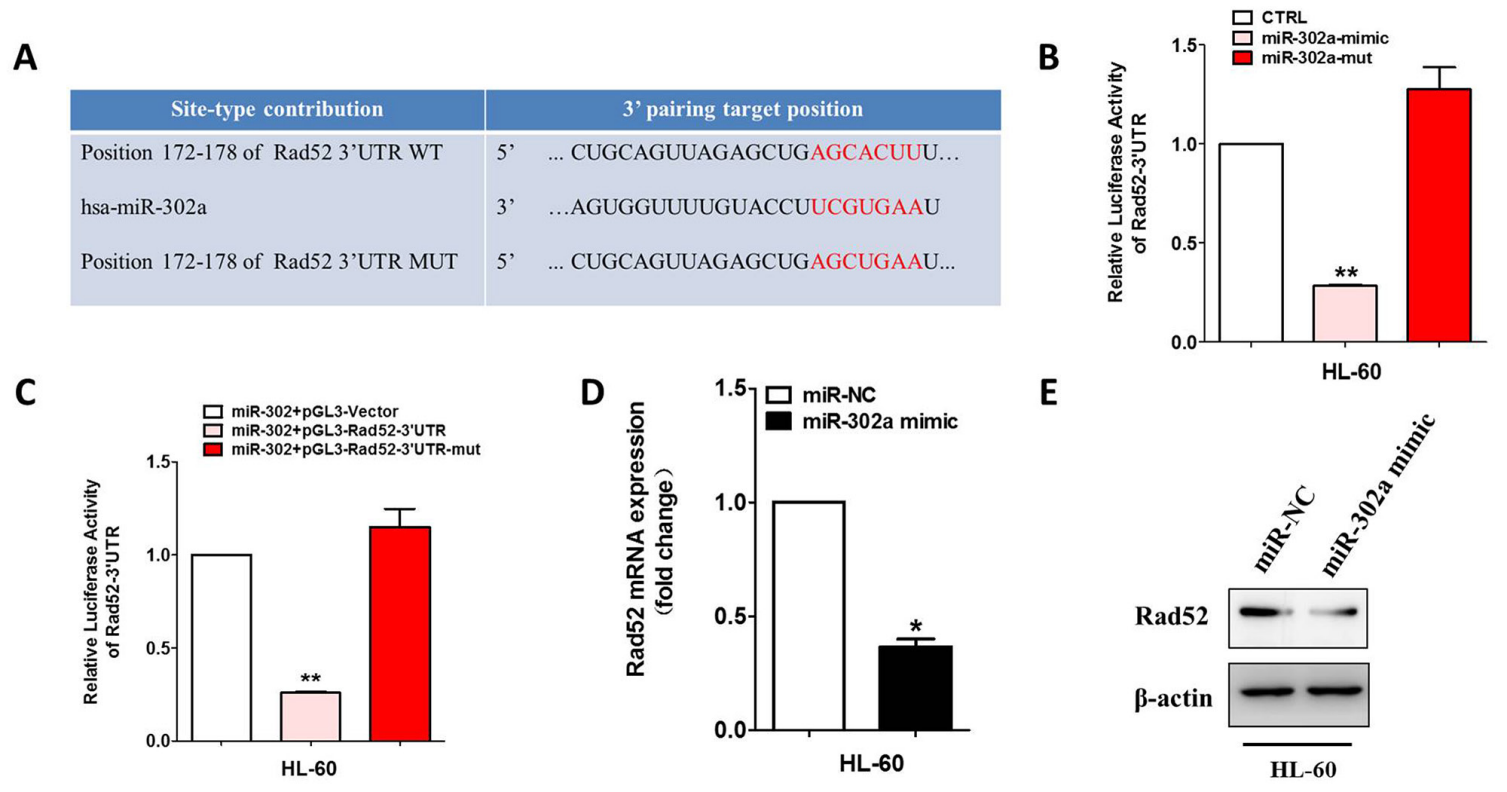
Rad52 is a direct target of miR-302a **(A)** Schematic representation of Rad52 3′-UTRs showing putative miR-302a target site. **(B)** the relative luciferase activity of miR-302a mimics or mutant with pGL3-Rad52 constructs in HL-60 cell lines. **(C)** the relative luciferase activity of Rad52 indicated constructs in HL-60 cell lines. **(D)** qRT-PCR was performed to detect the expression of Rad52 in HL-60 transfected with miR-302a mimics or negative control. **(E)** Western blot analysis of Rad52 expression in HL-60 cells transfected with negative control or miR-302. Each bar represents the mean of three independent experiments.* P<0.05, ** P<0.01.

To search for the miR-302a target gene involved in VP-16 sensitivity, we used several bioinformatics methods to help identify the target human genes of miR-302a. Among the targets predicted by the search programs of TargetScan, miRanda and miRDB, we found Rad52 was the theoretical target gene of miR-302a (Figure 3A). RAD52 3’UTR contains the complementary binding sites of miR-302a and it was cloned into a luciferase reporter vector to evaluate the influence of miR-302a on the expression of a reporter gene using a luciferase assay (Figure 3B and Supplementary Figure 2). We found that the expression of the reporter gene in the recombinant plasmid containing RAD52 3’UTR was significantly attenuated in miR-302a-overexpressing cells and could be restored in miR-302a-mutant cells. To validate that miR-302a can directly bind to and regulate the levels of Rad52 mRNA through the predicted binding sites, we altered bases of Rad52 in the putative miR-302a binding site and found that the mutant 3’UTRs were completely refractory to miR-302a-mediated luciferase reporter repression in HL-60 cells (Figure 3C and Supplementary Figure 2). This result was confirmed by qRT-PCR and Western blot analysis (Figure 3D, 3E and Supplementary Figure 2). All these data suggested that Rad52 is a direct target of miR-302a.

### Downregulation of Rad52 increased VP-16 sensitivity of the HL-60 cell

**Figure 4:**
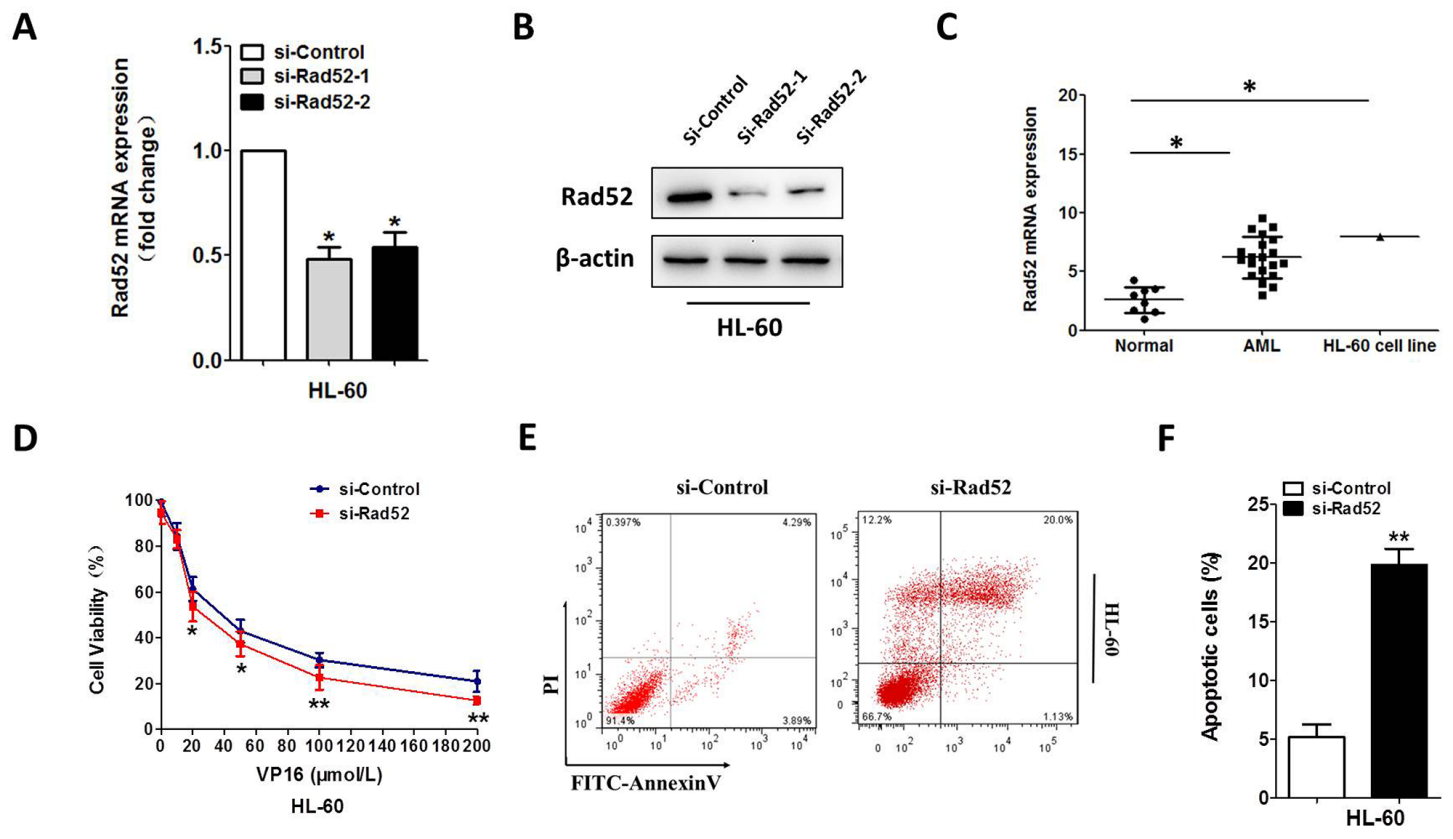
Downregulation of Rad52 increased VP-16 sensitivity of the HL-60 cell **(A,B)** downregulation of Rad52 in HL-60 transfected with Rad52 siRNA, control sequence served as loading control. qRT-PCR and Western blot were conducted to measure the Rad52 expression level. β-actin served as loading control. **(C)** qRT-PCR analysis of Rad52 mRNA expression in MNCs from healthy people, AML patients and AML cell line HL-60. **(D)** downregulation of Rad52 enhance the sensitivity to VP16 in HL-60 cell. **(E,F)** HL-60 cells were stained with PI and FITC-Annexin V, the percentage of apoptosis cell measured by flow cytometry. Each result represents the mean of three independent experiments.* P<0.05, ** P<0.01.

We continued to investigate whether Rad52 contributed to Vp-16 resistance of the HL-60 and U937 cells. We silenced Rad52 with siRNA, which prominently reduced endogenous Rad52 expression (Figure 4A, 4B and Supplementary Figure 3). We also investigated the mRNA expression in AML patients as well as controls. Rad52 were notably overexpressed in AML patients and AML cell lines with respect to controls (Figure 4C and Supplementary Figure 3). After 48h exposure to VP-16, proliferation and apoptosis assays were conducted. The HL-60 and U937 cells with Rad52 knockdown showed a much lower proliferation rate compared with the control groups (Figure 4D and Supplementary Figure 3). AnnexinV-FITC assays on VP-16-treatment cell line revealed a significant increase in VP-16-induced apoptosis after Rad52 silencing (Figure 4E, 4F and Supplementary Figure 3).

### MiR-302a sensitizes xenograft tumors to chemotherapeutic drug *in vivo*

**Figure 5:**
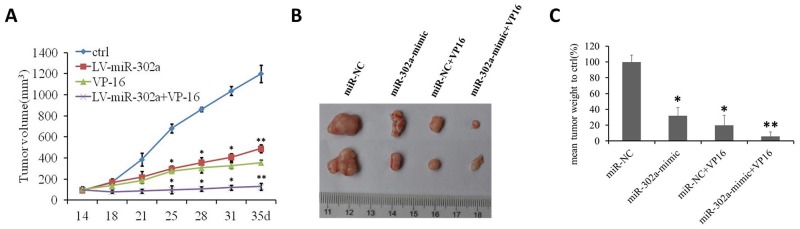
MiR-302a sensitizes xenograft tumors to a chemotherapeutic drug *in vivo* **(A)** *in vivo* growth rates of tumor volume of miR-NC, miR-302a, miR-NC+VP16 and miR-302a+VP-16 xenograft tumor grown in nude mice. **(B)** The representive picture and the mean weight of xenograft tumors. **(C)** the percentage of the mean weight to miR-NC (n=5 per group). * P<0.05, ** P<0.01.

To evaluate the effect of miR-302a on the sensitivity of AML cells to a chemotherapeutic drug (VP-16) *in vivo*, HL-60 and U937 cells stably overexpressing MSCV vector or MSCV-miR-302a were injected into the right flanks of nude mice. When the tumors were palpable, the MSCV and miR-302a mice were subdivided into two groups and treated with either vehicle (water) or VP-16 (20 mg/kg). Notably, within 3 weeks of treatment, miR-302a mice that received VP-16 had pronounced inhibition of tumor growth compared to MSCV mice that received the agent or miR-302a mice that received the vehicle control (Figure 5A–5C and Supplementary Figure 4). This indicates that miR-302a enhances the tumor-suppressive effect of VP-16 in a xenograft model of human AML.

### Downregulation of Rad52 by miR-302a suppresses cell proliferation and chemoresistance, in part by activating the AKT/Gsk-3β/β-catenin cascade

**Figure 6:**
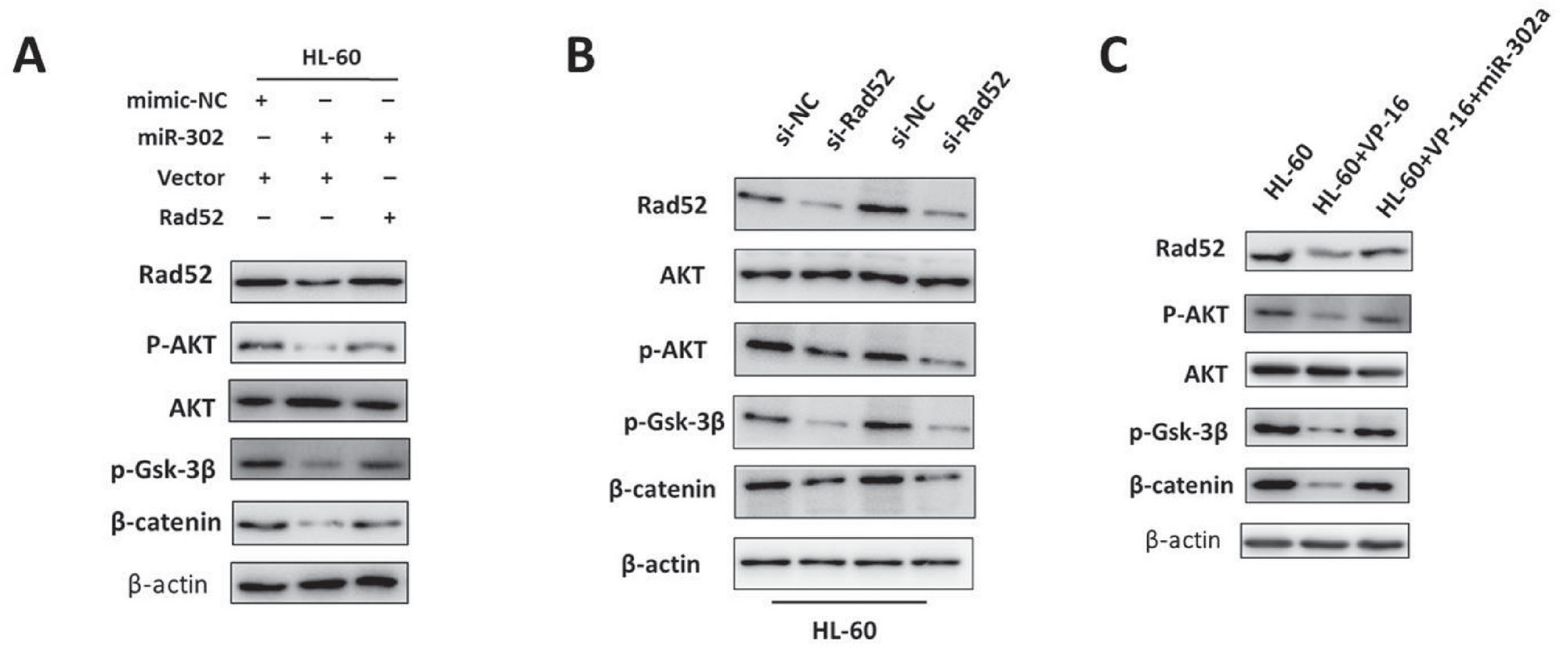
Downregulation of Rad52 by miR-302a suppresses cell proliferation and chemoresistance, in part by activating the AKT/Gsk-3β/β-catenin cascade **(A)** HL-60 cell lines were transfected with miR-302a or miR-302a and Rad52 overexpression vector respectively. The expression of Rad52, p-AKT, AKT, p-GSK3β, β-catenin were detected by Western blot. **(B)** Western blot analysis of Rad52, p-AKT, AKT, p-GSK3β, β-catenin expression in HL-60 cells transfected with negative control or Rad52 siRNA. **(C)** HL-60 cell lines treated with VP-16 or co-transfected with miR-302a mimics. The expression of Rad52, p-AKT, AKT, p-GSK3β, β-catenin were measured by Western blot. β-actin treated as loading control.

To identify the mechanisms implicated in VP-16 resistance, we examined the protein expression of several classical molecules that have been reported to regulate drug-induced apoptosis. We next examined the AKT and Wnt/β-catenin signaling pathway related proteins. Overexpression of miR-302a failed to reduce the total amount of AKT protein. However, the levels of endogenous Rad52, phospho-AKT, and phospho-Gsk-3β were reduced markedly under these conditions, and these changes facilitated β-catenin destruction (Figure 6A and Supplementary Figure 5A). In accordance with the above results, inhibition the expression of Rad52 downregulates the levels of phospho-AKT, phospho-Gsk-3β, β-catenin, c-myc and cyclin D1 in HL-60 and U937 cells (Figure 6B, Supplementary Figure 5B and Supplementary Figure 6). Next, we found that the AKT/β-catenin signaling was inactivated in VP-16 treatment cells, and that overexpression of miR-302a reversed the decreased expression of β-catenin by promoting Rad52/AKT/β-catenin signaling (Figure 6C and Supplementary Figure 5C). In addition, we investigated the expression levels of β-catenin target-genes after overexpression of miR-302a by western blotting assay (Supplementary Figure 6). Taken together, these results reveal that miR-302a might enhance VP-16 sensitivity by targeting Rad52 through regulating the AKT/Gsk3β/β-catenin pathway.

## DISCUSSION

The present *in vitro* and *in vivo* study assays presented herein demonstrate that miR-302a down-regulates the Rad52 gene expression. In this study, we showed that miR-302a was downregulated in VP-16-treated AML cells. Our results provide evidence that miR-302a synergistically increases the sensitivity of AML cells to VP-16 and demonstrate that miR-302a downregulates Rad52 to inhibit tumor growth and chemoresistance, in part via the AKT/Gsk-3β/β-catenin signaling cascade.

It is well known that miRNAs play vital role in various biological processes, including proliferation, cellular differentiation, signal transduction and carcino-genesis [17, 18, 19]. The data presented herein highlight the potential clinical utility of miR-302a levels as a valuable biomarker that reflects the expression of miR-302a in human AML samples. We found that miR-302a tuned-down both the proliferation and growth of AML cells. Here, in this research we identified for the first time that miR-302a exert effective functions on improving AML VP-16 sensitivity. The potential mechanism mediating these effects may be associated with the capacity of miR-302a to inhibit cell growth and induce cells apoptosis in HL-60 and U937 cells. To the best of our knowledge, this is the first study to demonstrate that miR-302a levels could be a valuable biomarker and an important prognostic factor for human AML. So far the precise mechanisms through which miRNAs get involved in AML VP-16 resistance remain unknown.

To investigate the underlying mechanisms by which miR-302a influence AML VP-16 sensitivity, we used bioinformatic methods to predict the possible target genes. As shown in Figure 3, Rad52 is the specific target of miR-302a. The Rad52 protein was a member homologous recombination (HR) and non-homologous end joining pathway that was important for DNA break repair and maintenance of genome integrity. VP-16 inhibited topoisomerase II activity to enhance cell apoptosis by making DNA break, Rad52 was a key protein in DNA break repair. As shown in Figures 4, 5 and Supplementary Figure 4, we prove downregulation of Rad52 increased VP-16 sensitivity of AML cell lines. As well, we found miR-302a sensitive AML cell lines to VP-16 in nude mice *in vivo*.

A serious miRNA, such as miR-374 [20], miR-203 [21], miR-21 [22], miR-7 [23, 24], play a role in cell proliferation, cancer metastasis, epithelial-mesenchymal transition et al through AKT/β-catenin signaling pathway. In the present study, miR-302a can suppress cell proliferation and decrease VP-16 resistance by activating AKT/β-catenin signaling.

In summary, our study have found miR-302a enhance the sensitivity of acute myeloid leukemia cell lines to VP-16 by targeting Rad52 and in part via the AKT/Gsk-3β/β-catenin signaling cascade. These findings proved miR-302a could serve as a therapeutic target or diagnostic/prognostic marker for acute myeloid leukemia therapy.

## MATERIALS AND METHODS

### Patient samples

Bone marrow (BM) samples were obtained from 20 AML patients and 8 healthy donors at Huai’an first people’s hospital, Nanjing Medical University. Written informed consent was obtained from each patient, and the use of clinical specimens was approved by the Institutional Ethics Committee. Mononuclear cells were isolated from the bone marrow samples using density-gradient centrifugation with Ficoll-Paque Plus (Ficoll, Pharmacia LKB Biotechnology, NY, USA) and stored at −80°C.

### Cell lines and cell culture

The human AML cell line HL-60 was purchased from the China Center for Type Culture Collection (CCTCC). The HL-60 cell was cultured by IMDM (Hyclone, USA) supplemented with 20% fetal bovine serum (Gibco, USA) in a 37°C humidified incubator with 5% CO_2_.

### Transfection

The synthetic mimics of miR-302a along with scrambled oligonucleotides were purchased from Genepharma (Shanghai, China). SiRNAs of Rad52 and its negative control were synthesized by Genscript (Nanjing, China). Lipofectamine 2000 reagent (Invitrogen) was used to transfect cells with miRNAs or siRNAs at a final concentration of 50 nM according to the manufacturer’s protocol.

And the sequences of miRNA and siRNA were as follows:

NC: AGGUAGUGUAAUCGCCUUG;
siRad52-1: UGAGAUGUUUGGUUACAAU;
siRad52-2: CCCUGAAGACAACCUUGAA;
miR-NC: UUGUACUACACAAAAGUACUG
miR-302a mimic: UAAGUGCUUCCAUGUUUUGGUGA.

### Proliferation and apoptosis assay

Viability was measured using cell counting kit-8 (beyotime, Shanghai, China) as described [25]. The apoptosis assay was conducted using Annexin V/propidium iodide (PI) Apoptosis Detection Kit (BD Biosciences) under thze guidence of the manufacturer’s instructions. Apoptosis rate of cells was measured by Becton Dickinson FACS Calibur flow cytometer, detecting the fluorescence of at least 10000 cells each sample.

### Luciferase reporter assay

The 3’UTR of Rad52 and mutated controls (position 172-178bp mutant into AGCUGAA) were cloned into the pGL3-basic Luciferase plasmid (Promega, USA). miRNA mimics and pRL-TK Renilla luciferase report plasmid were then transfected into the HL-60 and U937 cell containing wild-type or mutant 3’UTR pGL3-basic plasmids. pRL-TK Renilla luciferase report plasmid was used as internal loading control. After cell lysis the luciferase activity was measured using Luciferase Reporter Assay System (Promega Corporation Madison, WI, USA) according to manufacturer’s protocol.

### Quantitative real-time PCR

Total RNA was isolated from cells using Trizol reagent (Invitrogen, USA) following the manufacturer’s instructions. First strand cDNA synthesis was performed using a PrimeScript™ RT reagent Kit (TaKaRa, Dalian, China). The cDNA was amplified using an SYBR Premix Ex Taq™ II (TaKaRa, Dalian, China). PCR amplification was performed with the StepOnePlus™ real-time PCR machine. β-actin and U6 were used for normalization of mRNA and miRNA respectively. Relative quantification of mRNA expression levels was determined using the relative standard curve method following the manufacturer’s instructions. The relative expression of genes to internal control (β-actin or U6) was calculated using the 2^−(ΔΔCt)^ method. Primer sequences used in the experiments were as follows: Rad52 forward 5’- AGAAGGTTAAAGTCAATGG-3’, reverse 5’-ATCACGAAGATTGTTCTTC-3’; β-actin forward 5’- ATCACTGCCACCCAGAAGAC -3’, reverse 5′- TTTCTAGACG GCAGGTCAGG-3’;

### Western blot analysis

Cells were lysed in RIPA lysis buffer(50mM Tris(pH 7.4), 150mM NaCl, 1% NP-40, 0.5% sodium deoxycholate, 1mM PMSF and protease inhibitor cocktail) for 30min on ice, protein quantificated with BCA protein assay kit (Thermo Fisher). Cellular proteins were extracted and separated in 4-10% Tris glycine/SDS-polyacrylamide gels and electrotransferred to ECL nitrocellulose membranes. The membranes were blocked with 5% nonfat milk and incubated with specific antibodies. Bands were detected using the ECL chemiluminescence detection method (Millipore) and exposure on Tanon image system (Shanghai, China). The antibody used in the experiment: Rad52 (#3425); p-AKT (#4060); AKT (#4691); GSK-3β(#5558); β-catenin (#8480); β-actin (#3700); H3(#4499); c-myc(ab32072); cyclinD1(ab1663). All of the antibodies were purchased from Cell Signaling Technology Company and Abcam. The β-actin protein was used as the endogenous control.

### Xenograft studies

Twenty nude mice (BALB/c-nu; 5 weeks old) were divided randomly into two cohorts and inoculated in the right flank with 2x 10^6^ HL-60 cells that stably expressed MSCV vector or MSCV-miR-302a. Once palpable tumors formed, each cohort was treated with the VP-16 (20mg/kg). Treatments were administered intraperitoneally twice a week and tumors were measured every 3-4 days using a digital caliper. After 5 weeks of treatment, the mice were sacrificed humanely and the tumors were weighed. The animal protocol used in this study was reviewed and approved by the Huai’an first people’s hospital, Nanjing Medical University Animal Care and Use Committee for ethical procedures and scientific care.

### Statistical analysis

Group comparisons of normally distributed data were performed using t-tests (two sample) or one-way ANOVA (multiple comparisons). The non-parametric Wilcoxon test was used to analyze continuous non-normally distributed data. For multiple comparisons, the Tukey Kramer honestly significant difference was applied following ANOVA. Categorical variables were compared using χ 2 analysis or Fisher’s exact test. *P < 0.05* (two-tailed) was considered significance.

## SUPPLEMENTARY MATERIALS FIGURES



## References

[1] Döhner H, Weisdorf DJ, Bloomfield CD (2015). Acute Myeloid Leukemia. N Engl J Med.

[2] Saultz JN, Garzon R (2016). Acute Myeloid Leukemia: A Concise Review. J Clin Med.

[3] Jensen PB, Sehested M (1997). DNA topoisomerase II rescue by catalytic inhibitors: a new strategy to improve the antitumor selectivity of etoposide. Biochem Pharmacol.

[4] Hande KR (1998). Etoposide: four decades of development of a topoisomerase II inhibitor. Eur J Cancer.

[5] Baldwin EL, Osheroff N (2005). Etoposide, topoisomerase II and cancer. Curr Med Chem Anticancer Agents.

[6] Meresse P, Dechaux E, Monneret C, Bertounesque E (2004). Etoposide: discovery and medicinal chemistry. Curr Med Chem.

[7] Montecucco A, Biamonti G (2007). Cellular response to etoposide treatment. Cancer Lett.

[8] Oom AL, Humphries BA, Yang C (2014). MicroRNAs: novel players in cancer diagnosis and therapies. BioMed Res Int.

[9] Garzon R, Volinia S, Liu CG, Fernandez-Cymering C, Palumbo T, Pichiorri F, Fabbri M, Coombes K, Alder H, Nakamura T, Flomenberg N, Marcucci G, Calin GA (2008). MicroRNA signatures associated with cytogenetics and prognosis in acute myeloid leukemia. Blood.

[10] Card DA, Hebbar PB, Li L, Trotter KW, Komatsu Y, Mishina Y, Archer TK (2008). Oct4/Sox2-regulated miR-302 targets cyclin D1 in human embryonic stem cells. Mol Cell Biol.

[11] Fareh M, Turchi L, Virolle V, Debruyne D, Almairac F, de-la-Forest Divonne S, Paquis P, Preynat-Seauve O, Krause KH, Chneiweiss H, Virolle T (2012). The miR 302-367 cluster drastically affects self-renewal and infiltration properties of glioma-initiating cells through CXCR4 repression and consequent disruption of the SHH-GLI-NANOG network. Cell Death Differ.

[12] Rosa A, Brivanlou AH (2011). A regulatory circuitry comprised of miR-302 and the transcription factors OCT4 and NR2F2 regulates human embryonic stem cell differentiation. EMBO J.

[13] Liang Z, Ahn J, Guo D, Votaw JR, Shim H (2013). MicroRNA-302 replacement therapy sensitizes breast cancer cells to ionizing radiation. Pharm Res.

[14] New JH, Sugiyama T, Zaitseva E, Kowalczykowski SC (1998). Rad52 protein stimulates DNA strand exchange by Rad51 and replication protein A.. Nature.

[15] Shinohara A, Ogawa T (1998). Stimulation by Rad52 of yeast Rad51-mediated recombination. Nature.

[16] Hu H, Gatti RA (2011). MicroRNAs: new players in the DNA damage response. J Mol Cell Biol.

[17] Bartel DP (2004). MicroRNAs: genomics, biogenesis, mechanism, and function. Cell.

[18] Hammond SM (2006). MicroRNA therapeutics: a new niche for antisense nucleic acids. Trends Mol Med.

[19] Krützfeldt J, Poy MN, Stoffel M

[20] Cai J, Guan H, Fang L, Yang Y, Zhu X, Yuan J, Wu J, Li M (2013). MicroRNA-374a activates Wnt/β-catenin signaling to promote breast cancer metastasis. J Clin Invest.

[21] Saini S, Arora S, Majid S, Shahryari V, Chen Y, Deng G, Yamamura S, Ueno K, Dahiya R (2011). Curcumin modulates microRNA-203-mediated regulation of the Src-Akt axis in bladder cancer. Cancer Prev Res (Phila).

[22] Sayed D, He M, Hong C, Gao S, Rane S, Yang Z, Abdellatif M (2010). MicroRNA-21 is a downstream effector of AKT that mediates its antiapoptotic effects via suppression of Fas ligand. J Biol Chem.

[23] Kefas B, Godlewski J, Comeau L, Li Y, Abounader R, Hawkinson M, Lee J, Fine H, Chiocca EA, Lawler S, Purow B (2008). microRNA-7 inhibits the epidermal growth factor receptor and the Akt pathway and is down-regulated in glioblastoma. Cancer Res.

[24] Zhou X, Hu Y, Dai L, Wang Y, Zhou J, Wang W, Di W, Qiu L (2014). MicroRNA-7 inhibits tumor metastasis and reverses epithelial-mesenchymal transition through AKT/ERK1/2 inactivation by targeting EGFR in epithelial ovarian cancer. PLoS One.

[25] Sun W, Shen W, Yang S, Hu F, Li H, Zhu TH (2010). miR-223 and miR-142 attenuate hematopoietic cell proliferation, and miR-223 positively regulates miR-142 through LMO2 isoforms and CEBP-β. Cell Res.

