# Supplementary material for: MiR-302a sensitizes leukemia cells to etoposide by targeting Rad52
Source: Oncotarget. 2017 May 16;8(43):73884–91. doi: 10.18632/oncotarget.17878 (PMC5650309; doi:10.18632/oncotarget.17878)
Supplement: Supplementary file 1 [file oncotarget-08-73884-s001.pdf]

MiR-302a sensitizes leukemia cells to etoposide by targeting Rad52

SUPPLEMENTARY MATERIALS

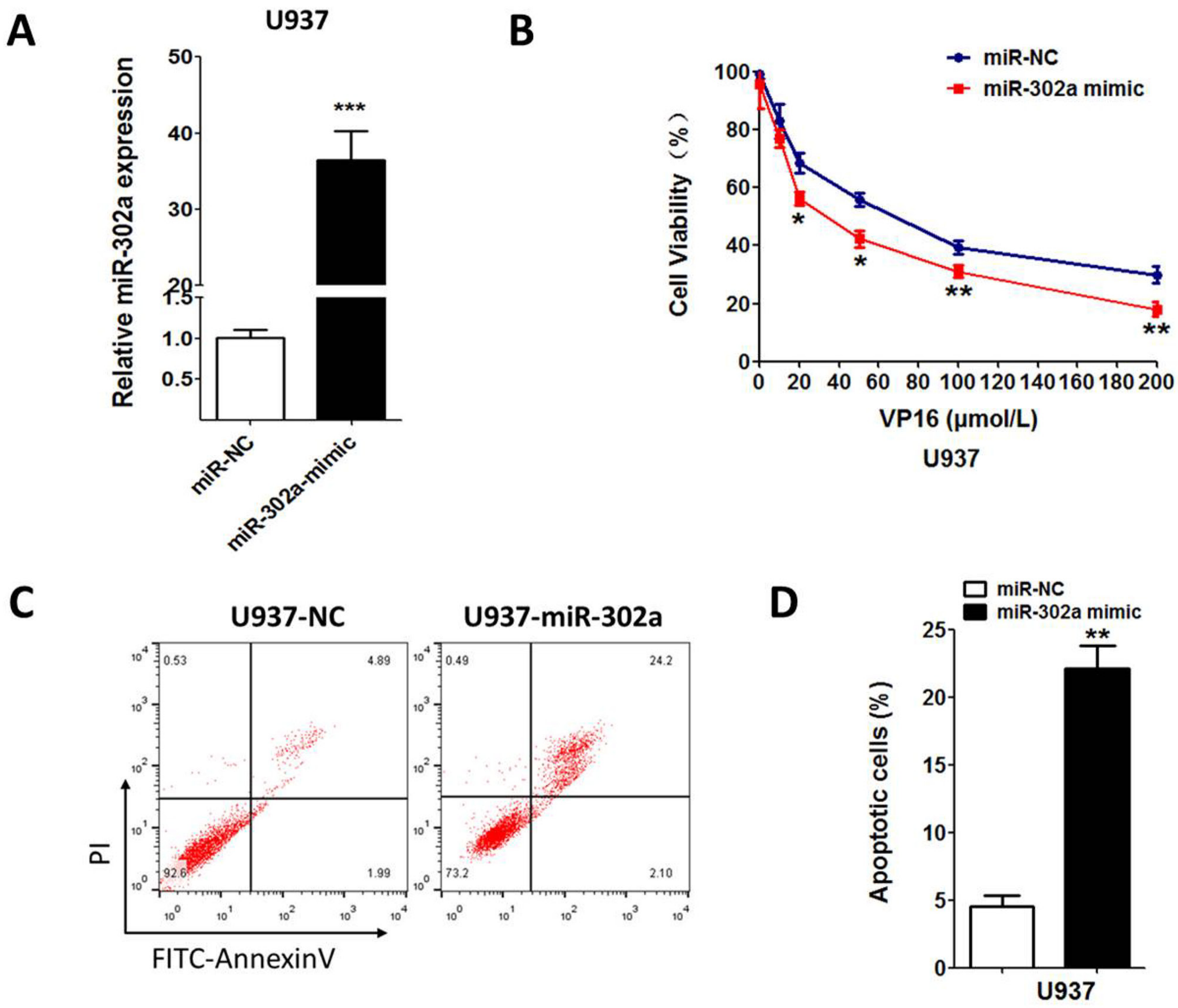

Supplementary Figure 1: Overexpression of miR-302a enhances the sensitivity to VP-16 in U937 Cell. **(A)** U937 was transfected the miR-302a or negative control by Lipo2000. the expression level of miR-302a was measured by qRT-PCR. **(B)** compare to negative control, overexpression of miR-302a enhance the sensitivity to VP16 measured by MTT in U937. **(C,D)** cells were stained with PI and FITC-Annexin V, the percentage of apoptosis cell measured by flow cytometry. Each result represents the mean of three independent experiments.* $P<0.05$, ** $P<0.01$. *** $P<0.001$.

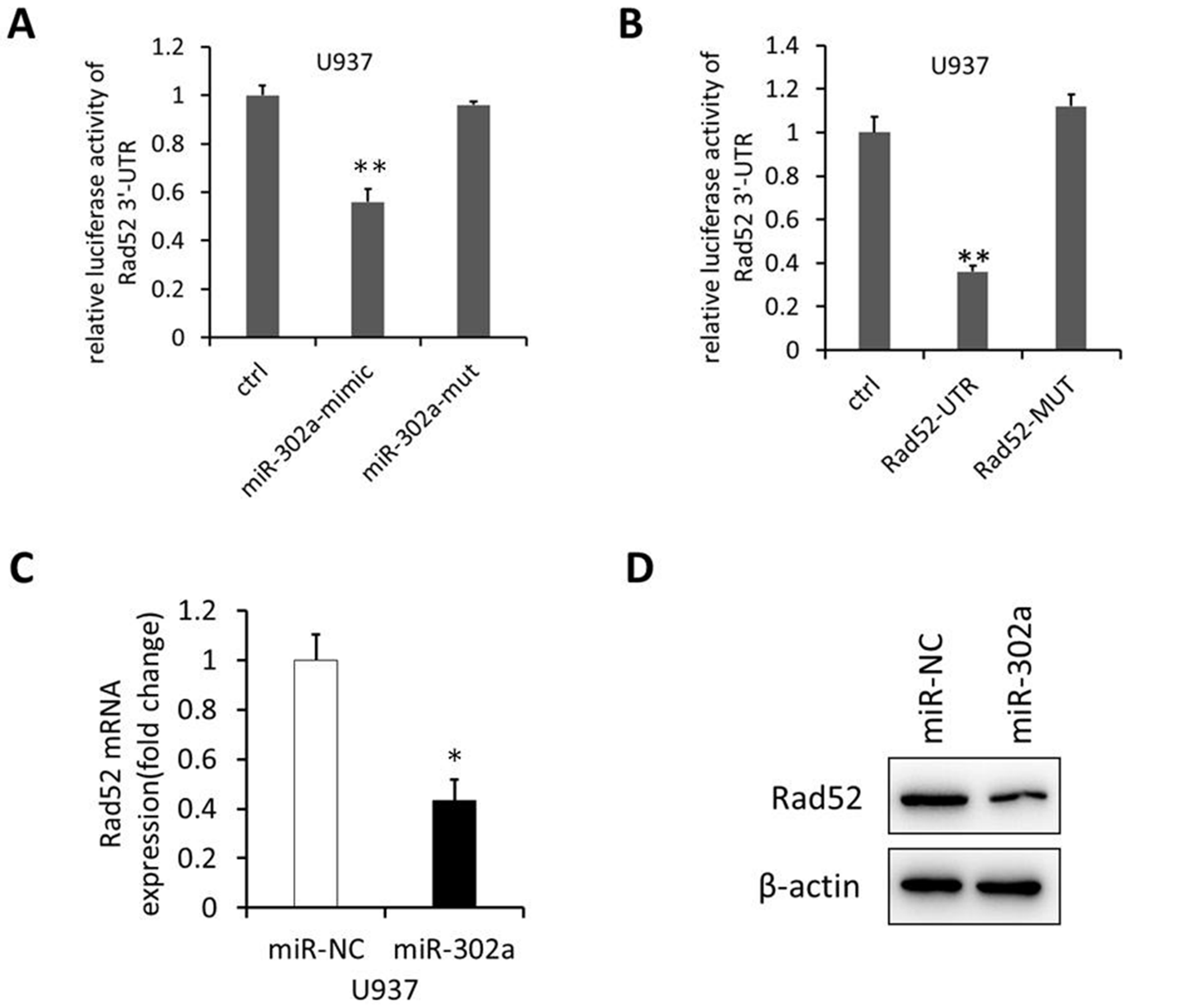

Supplementary Figure 2: Rad52 is a direct target of miR-302a. (A) the relative luciferase activity of miR-302a mimics or mutant with pGL3-Rad52 constructs in U937 cell lines. **(B)** the relative luciferase activity of Rad52 indicated constructs in U937 cell lines. **(C)** qRT-PCR was performed to detect the expression of Rad52 in U937 transfected with miR-302a mimics or negative control. **(D)** Western blot analysis of Rad52 expression in U937 cells transfected with negative control or miR-302. Each bar represents the mean of three independent experiments.* $P<0.05$, ** $P<0.01$.

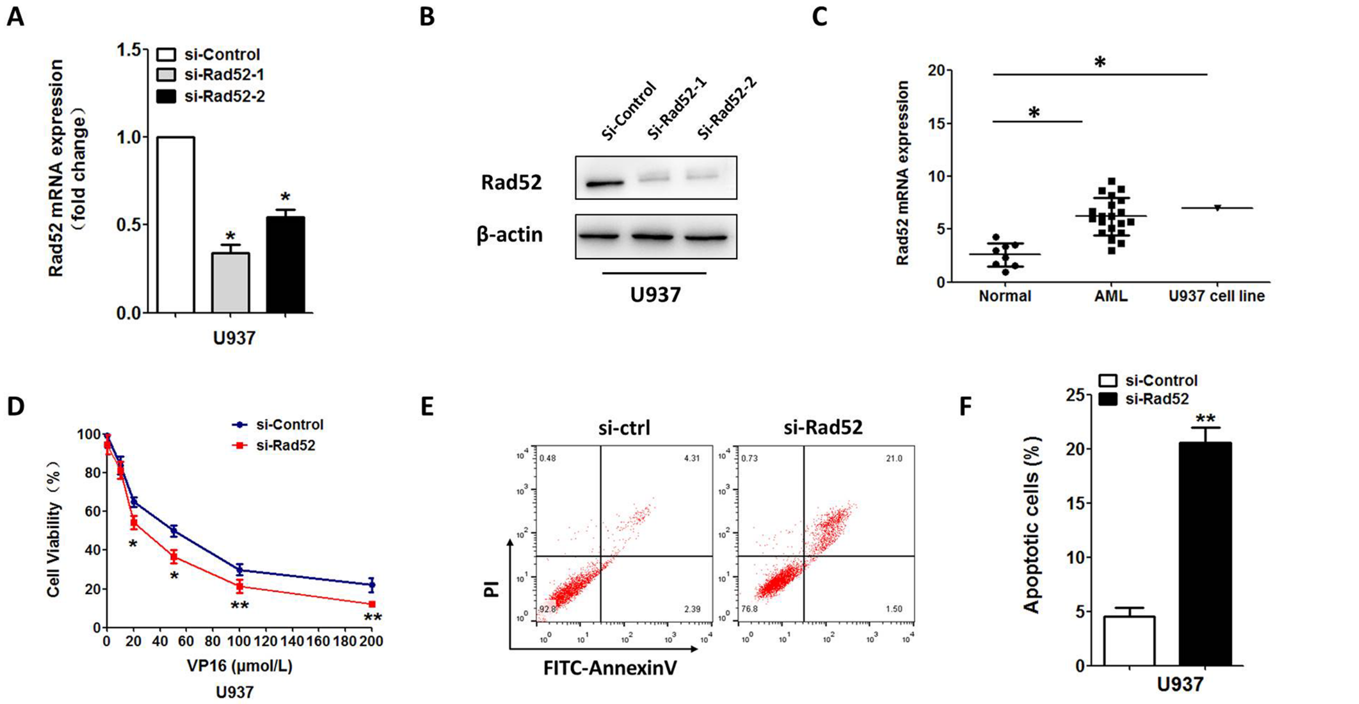

Supplementary Figure 3: Downregulation of Rad52 increased VP-16 sensitivity of the U937 cell. **(A,B)** downregulation of Rad52 in U937 transfected with Rad52 siRNA, control sequence served as loading control. qRT-PCR and Western blot were conducted to measure the Rad52 expression level. β-actin served as loading control. **(C)** qRT-PCR analysis of Rad52 mRNA expression in MNCs from healthy people, AML patients and AML cell line U937. **(D)** downregulation of Rad52 enhance the sensitivity to VP16 in U937 cell. **(E,F)** U937 cells were stained with PI and FITC-Annexin V, the percentage of apoptosis cell measured by flow cytometry. Each result represents the mean of three independent experiments.* P<0.05, ** P<0.01.

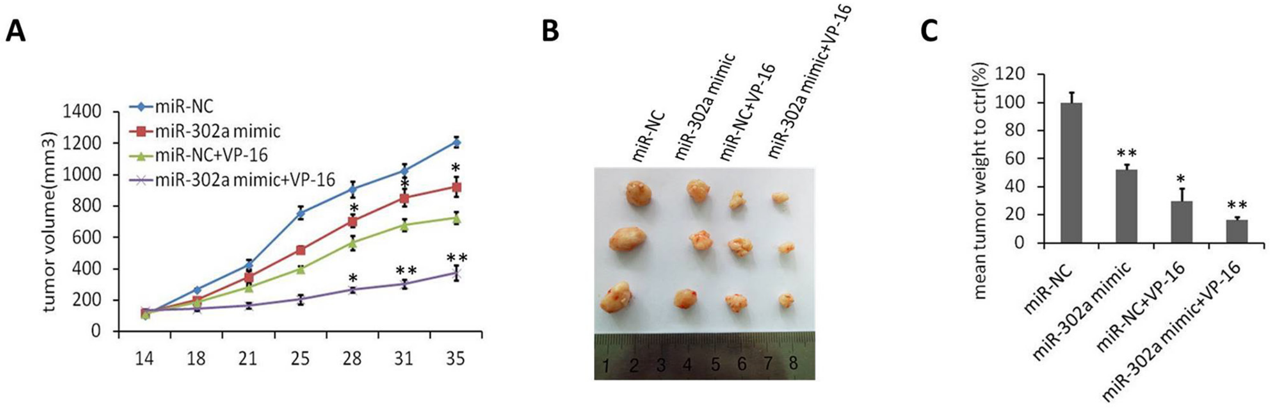

Supplementary Figure 4: MiR-302a sensitizes xenograft tumors to a chemotherapeutic drug *in vivo*. **(A)** *in vivo* growth rates of tumor volume of miR-NC, miR-302a, miR-NC+VP16 and miR-302a+VP-16 xenograft tumor grown in nude mice. **(B,C)** The representive picture and the mean weight of xenograft tumors. (n=5 per group) * P<0.05, ** P<0.01.

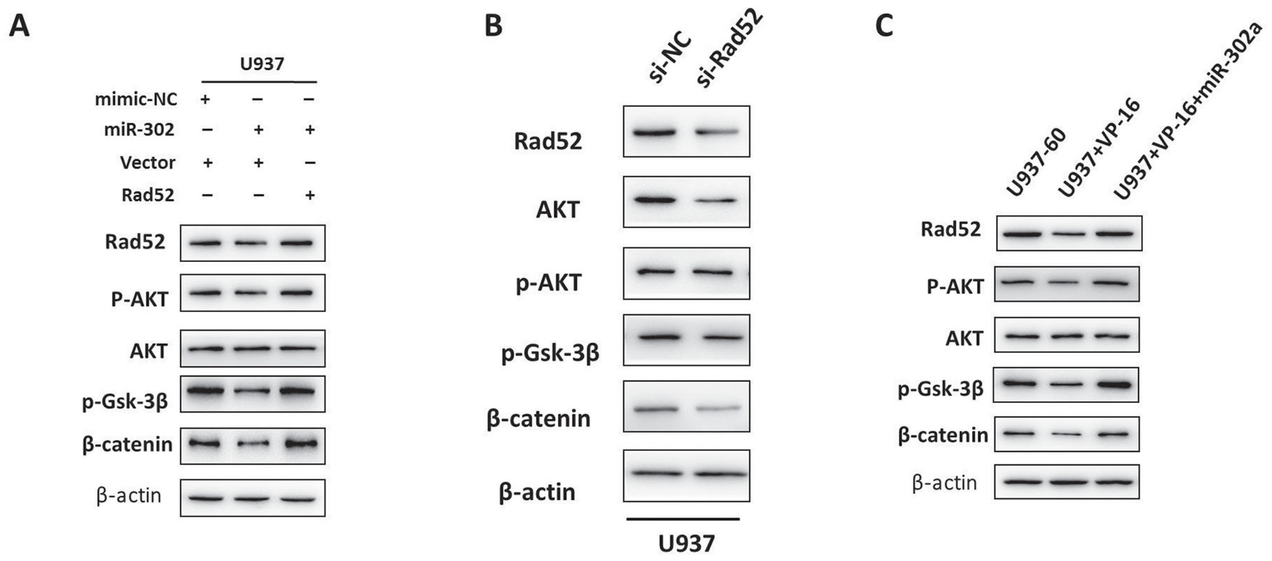

Supplementary Figure 5: Downregulation of Rad52 by miR-302a suppresses cell proliferation and chemoresistance, in part by activating the AKT/Gsk-3β/β-catenin cascade. **(A)** U937 cell lines were transfected with miR-302a or miR-302a and Rad52 overexpression vector respectively. The expression of Rad52, p-AKT, AKT, p-GSK3β, β-catenin were detected by Western blot. **(B)** Western blot analysis of Rad52, p-AKT, AKT, p-GSK3β, β-catenin expression in U937 cells transfected with negative control or Rad52 siRNA. **(C)** U937 cell lines treated with VP-16 or co-transfected with miR-302a mimics. The expression of Rad52, p-AKT, AKT, p-GSK3β, β-catenin were measured by Western blot. β-actin treated as loading control.

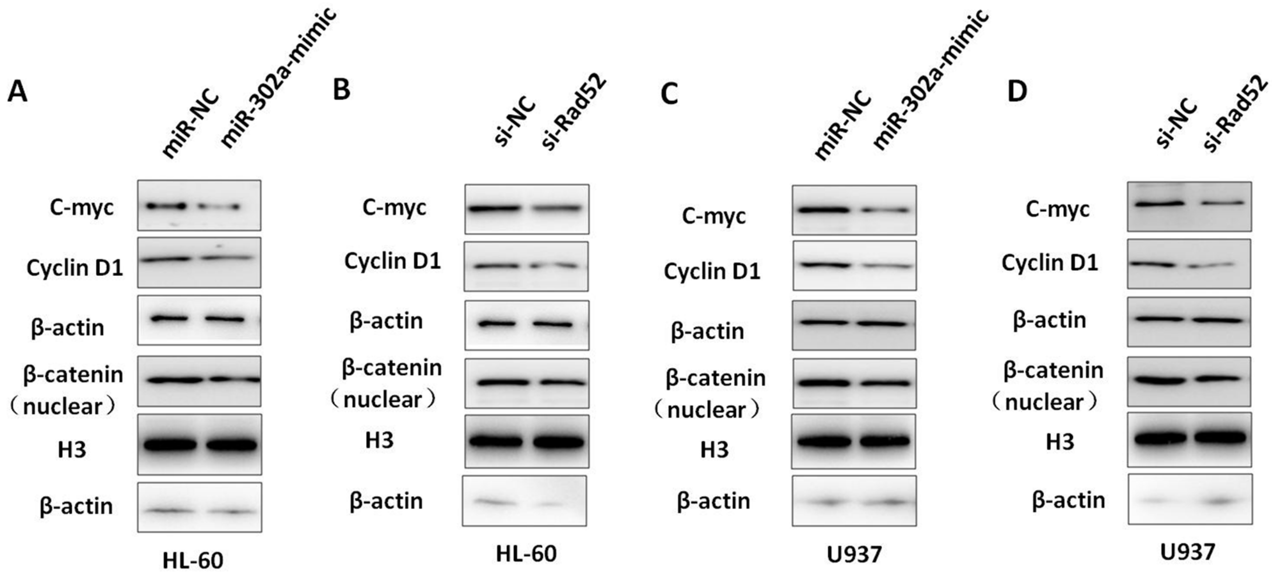

Supplementary Figure 6: Expression of β-catenin target-genes after knocking down Rad52 and overexpression of miR-302a in HL-60 and U937 cells. **(A,B)** HL-60 cell lines were transfected with miR-302a mimic or Rad52 siRNA respectively. The expression of C-myc, Cyclin D1, β-catenin (nuclear) were detected by Western blot. **(C,D)** U937 cell lines were transfected with miR-302a mimic or Rad52 siRNA respectively. The expression of C-myc, Cyclin D1, β-catenin (nuclear) were detected by Western blot. β-actin and H3 treated as loading control.
